# Supplementary figures and images for: Identification of OTUD6B as a new biomarker for prognosis and immunotherapy by pan-cancer analysis
Source: Front Immunol. 2022 Aug 16;13:955091. doi: 10.3389/fimmu.2022.955091 (PMC9425067; doi:10.3389/fimmu.2022.955091)

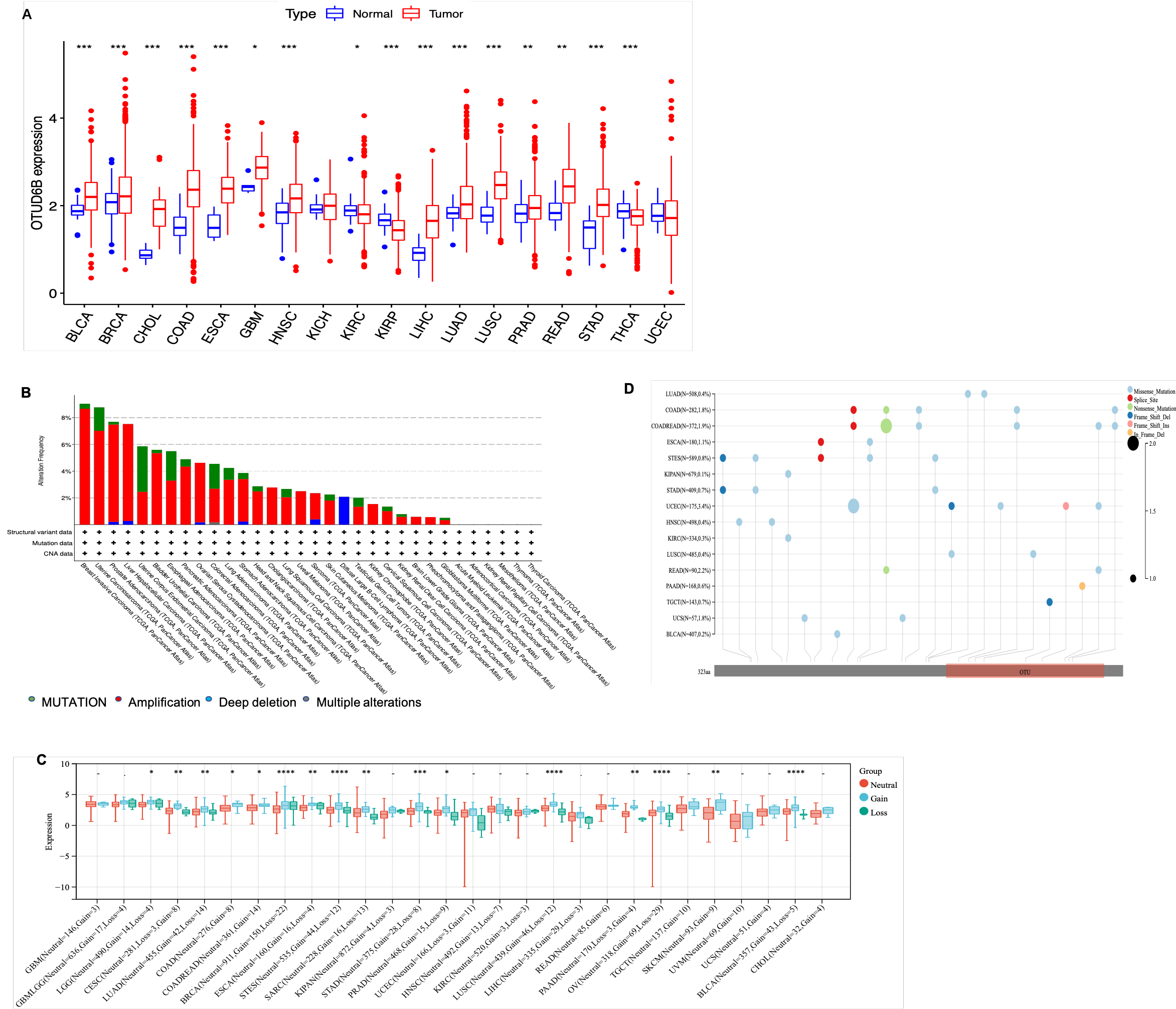

Supplement: Supplementary Figure 1 — Gene mutation profiles of OTUD6B in pan-cancer. (A) The expression differences of OTUD6B between normal and tumor tissues from TCGA database. (B) Gene alteration (C) Copy number variation of OTUD6B expression. (D) Mutant landscape of OTUD6B. [file Image_1.tiff]

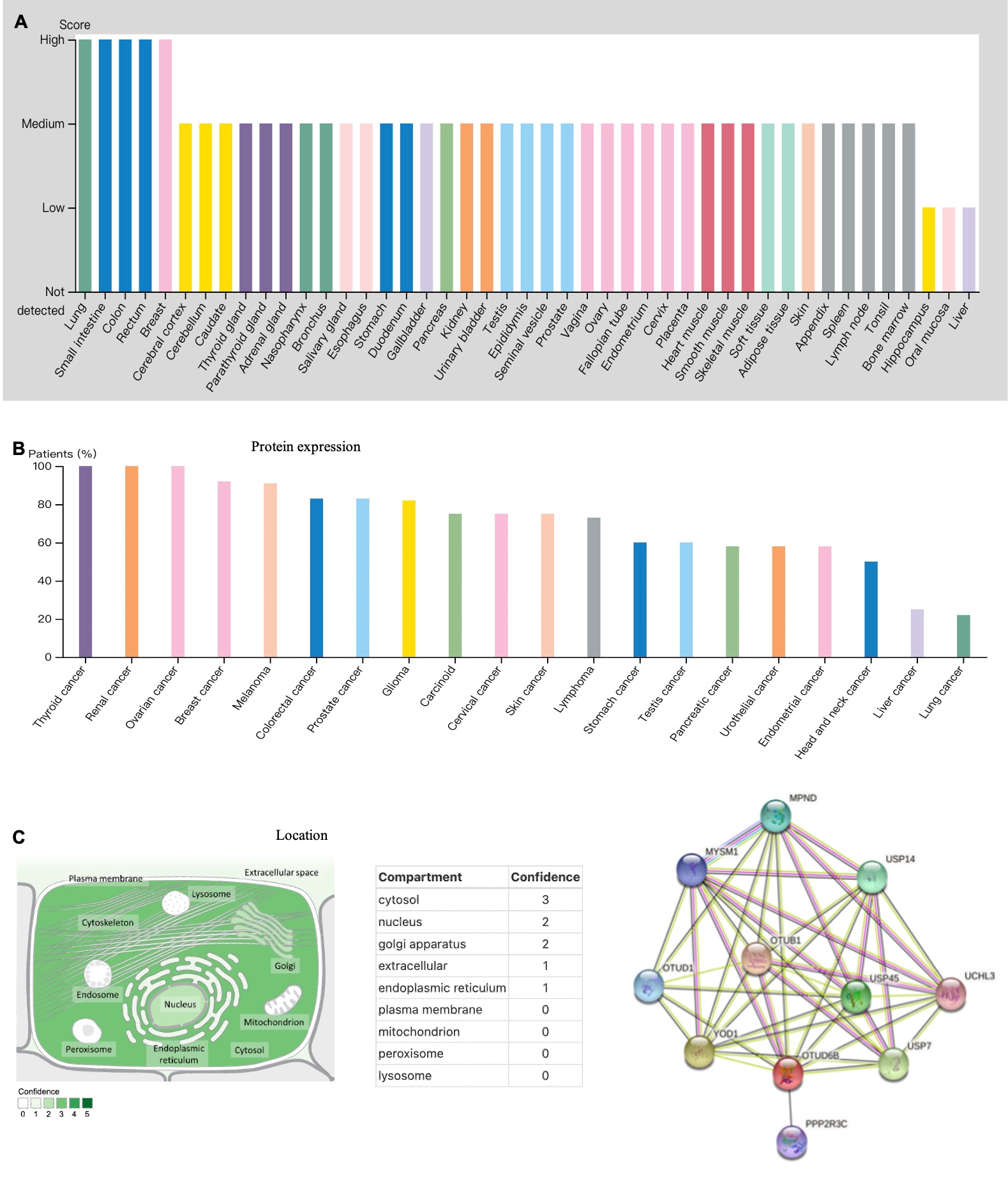

Supplement: Supplementary Figure 2 — Protein expression and location of OTUD6B in normal and tumor tissues. [file Image_2.jpeg]

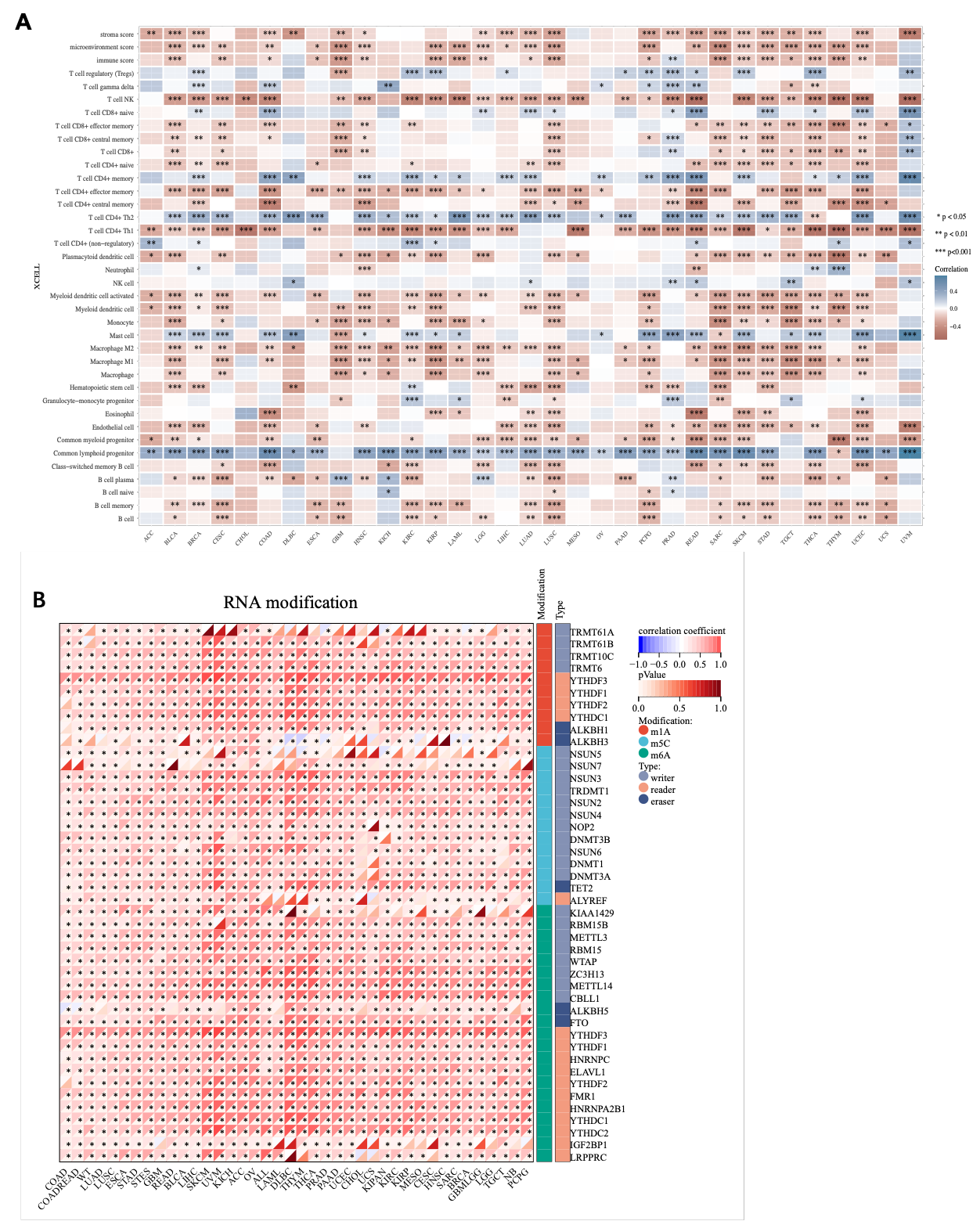

Supplement: Supplementary Figure 3 — (A) Correlation between OTUD6B expression and different immune cells using data from XCell database. (B) RNA modification and OTUD6B level in multiple tumor tissues. [file Image_3.tiff]

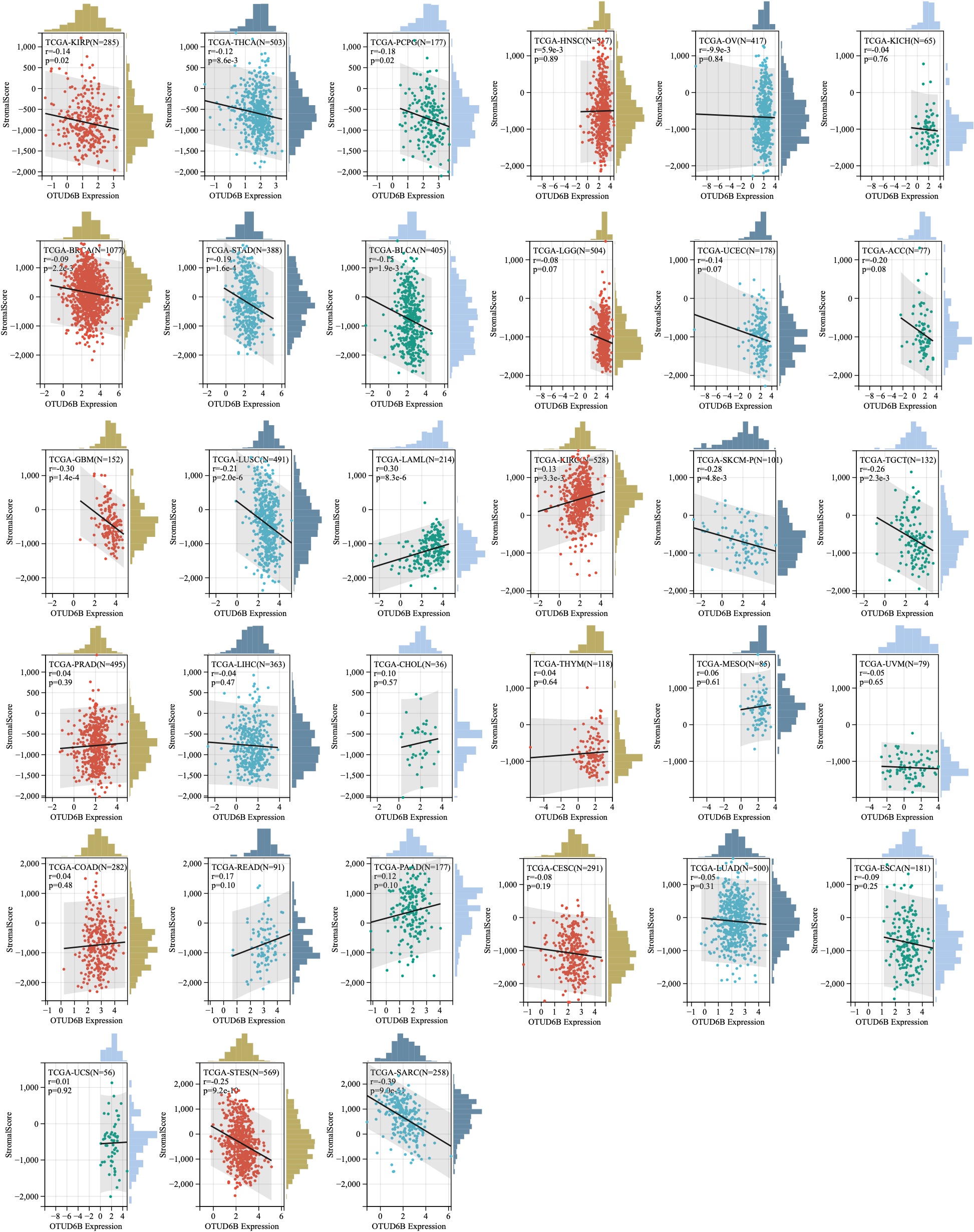

Supplement: Supplementary Figure 4 — Association of OTUD6B expression level with Stromal Score across different types of cancers. [file Image_4.jpeg]
